# The impact of HIV/SRH service integration on workload: analysis from the Integra Initiative in two African settings

**DOI:** 10.1186/1478-4491-12-42

**Published:** 2014-08-07

**Authors:** Sedona Sweeney, Carol Dayo Obure, Fern Terris-Prestholt, Vanessa Darsamo, Christine Michaels-Igbokwe, Esther Muketo, Zelda Nhlabatsi, Charlotte Warren, Susannah Mayhew, Charlotte Watts, Anna Vassall

**Affiliations:** 1 London School of Hygiene & Tropical Medicine, 15-17 Tavistock Place, London WC1H 9SH, UK; 2 Population Council, Washington, DC, USA; 3 Family Health Options Kenya, Nairobi, Kenya; 4 Family Life Association of Swaziland, Manzini, Swaziland

**Keywords:** Integration, HIV, SRH, Human resources, Staff time, Economics

## Abstract

**Background:**

There is growing interest in integration of HIV and sexual and reproductive health (SRH) services as a way to improve the efficiency of human resources (HR) for health in low- and middle-income countries. Although this is supported by a wealth of evidence on the acceptability and clinical effectiveness of service integration, there is little evidence on whether staff in general health services can easily absorb HIV services.

**Methods:**

We conducted a descriptive analysis of HR integration through task shifting/sharing and staff workload in the context of the Integra Initiative - a large-scale five-year evaluation of HIV/SRH integration. We describe the level, characteristics and changes in HR integration in the context of wider efforts to integrate HIV/SRH, and explore the impact of HR integration on staff workload.

**Results:**

Improvements in the range of services provided by staff (HR integration) were more likely to be achieved in facilities which also improved other elements of integration. While there was no overall relationship between integration and workload at the facility level, HIV/SRH integration may be most influential on staff workload for provider-initiated HIV testing and counselling (PITC) and postnatal care (PNC) services, particularly where HIV care and treatment services are being supported with extra SRH/HIV staffing. Our findings therefore suggest that there may be potential for further efficiency gains through integration, but overall the pace of improvement is slow.

**Conclusions:**

This descriptive analysis explores the effect of HIV/SRH integration on staff workload through economies of scale and scope in high- and medium-HIV prevalence settings. We find some evidence to suggest that there is potential to improve productivity through integration, but, at the same time, significant challenges are being faced, with the pace of productivity gain slow. We recommend that efforts to implement integration are assessed in the broader context of HR planning to ensure that neither staff nor patients are negatively impacted by integration policy.

## Introduction

The current crisis in human resources (HR) for health in many low- and middle-income countries raises uncertainty about how international goals for scale-up of HIV-related services can be met [1-4]. At the workforce level, there is an absolute shortage of qualified staff, leading to great need for efficiency improvements in HR utilization. This absolute shortage is often further exacerbated by inequitable distribution of health workers, causing some existing staff to become overworked while in other areas there may be excess capacity at the provider level. In light of this situation, integration of health services through task sharing has been suggested as a way to reduce the burden on HR for HIV testing, care and treatment services [5-7].

Considerations for integration come in the context of a wealth of existing evidence on the clinical and public health benefits of integration [8-10], its acceptability to both patients and providers [11-13], and emerging evidence that it may have an impact on cost more broadly [14-16]. One of the over-arching HR policy rationales for integrating services stems from the aim to improve the efficiency of HR use. This, in turn, is driven by an assumption that existing staff in maternal and child health (MCH) departments can easily absorb additional HIV-related activities, thereby improving the cost-effectiveness of service provision and reducing the need for additional dedicated HIV staff [17-19]. The integration of some related administrative tasks, such as taking patient details, may also have the potential to improve the efficiency of HR use by reducing the staff time required per patient visit. In some cases, where staff members have excess down time, offering additional services through integration may also lead to an increase in staff productivity.

However, to date there is very little evidence available concerning the feasibility of integrated service delivery, or on the impact on imbalances in staff workload when integration is implemented at scale. Where staff are already overworked, taking on additional tasks may lead to burnout or job dissatisfaction [11,20], thus reducing service quality. The objective of this paper is, therefore, to explore the relationship between HR integration (defined as increasing the range of services provided by individual staff members) and workload in an integrated HIV/sexual and reproductive health (SRH) service delivery setting, in order to inform the policy debates on integration. We describe below the level, characteristics and changes in HR integration in the context of other elements of HIV/SRH service integration in two African settings, and explore the impact of HR integration on staff workload and productivity.

## Methods

### Study setting

This work is carried out as part of the Integra Initiative - a large-scale study evaluating HIV/SRH integration over a five-year period [21]. The Integra Initiative (Integra) is one of the largest studies evaluating service integration to date, including twenty-four public and six private health facilities in Kenya, and eight public and two private health facilities in Swaziland. Study sites were chosen purposively, with an effort made to include a range of different settings, facility types, ownership and models of integration. The focus of Integra was to evaluate the effects of adding ‘non-core’ SRH and HIV services into the MCH unit. Core MCH services are defined as those which are consistently offered within the standard care package across all health facilities and include family planning (FP), postnatal care (PNC), and antenatal care (ANC). Non-core services are HIV/sexually transmitted infections (HIV/STI) services not consistently offered within MCH departments, including STI management (STI), HIV Counselling and testing (including voluntary HIV counselling and testing (VCT) and provider-initiated HIV testing and counselling (PITC)), cervical cancer screening (CaCx), CD4 count testing services, and antiretroviral therapy (ART). Data for this study was collected as part of a broader effort to evaluate changes in service costs (Obure CD, Sweeney S, Guinness L, Watts C, Integra Research Team, Vassall A: The costs of delivering integrated HIV and sexual reproductive health services in Kenya and Swaziland: a descriptive analysis, in preparation), and was collected for the financial years 2008 to 2009 (baseline), and 2010 to 2011 (endline).

### Conceptual and analytical framework

While integration can be defined as an increase in the range of services available to clients either within a department or at the facility level, as noted by Atun *et al*. [22] health services integration is not binary but can be better described as a continuum of coordination and collaboration and can include consolidating a number of processes and resources between services - including procurement processes, data collection and analysis, human resources, and physical space or infrastructure.

Integra was implemented as a programmatic intervention in facilities with a range of services offered. Integration was implemented in different ways across the sites, depending on the baseline circumstances of the facility and its ability to adapt. In the interest of reflecting this complexity of integration as implemented in a ‘real world’ setting, we adopted four broad measures of integration which could be evaluated in all sites, with a focus on providing any combination of ‘core’ and ‘non-core’ reproductive health/HIV services. These include joint utilization of human and physical resources, and expansion of the services available at the MCH level and at the facility level. In the context of HR, we define ‘HR integration’ as the provision of multiple services by one staff member. This can be realized either through moving services from a stand-alone department to one providing both SRH and HIV services, or through adding services to the basic package offered by a staff member without dropping their pre-existing tasks.

**Figure 1:**
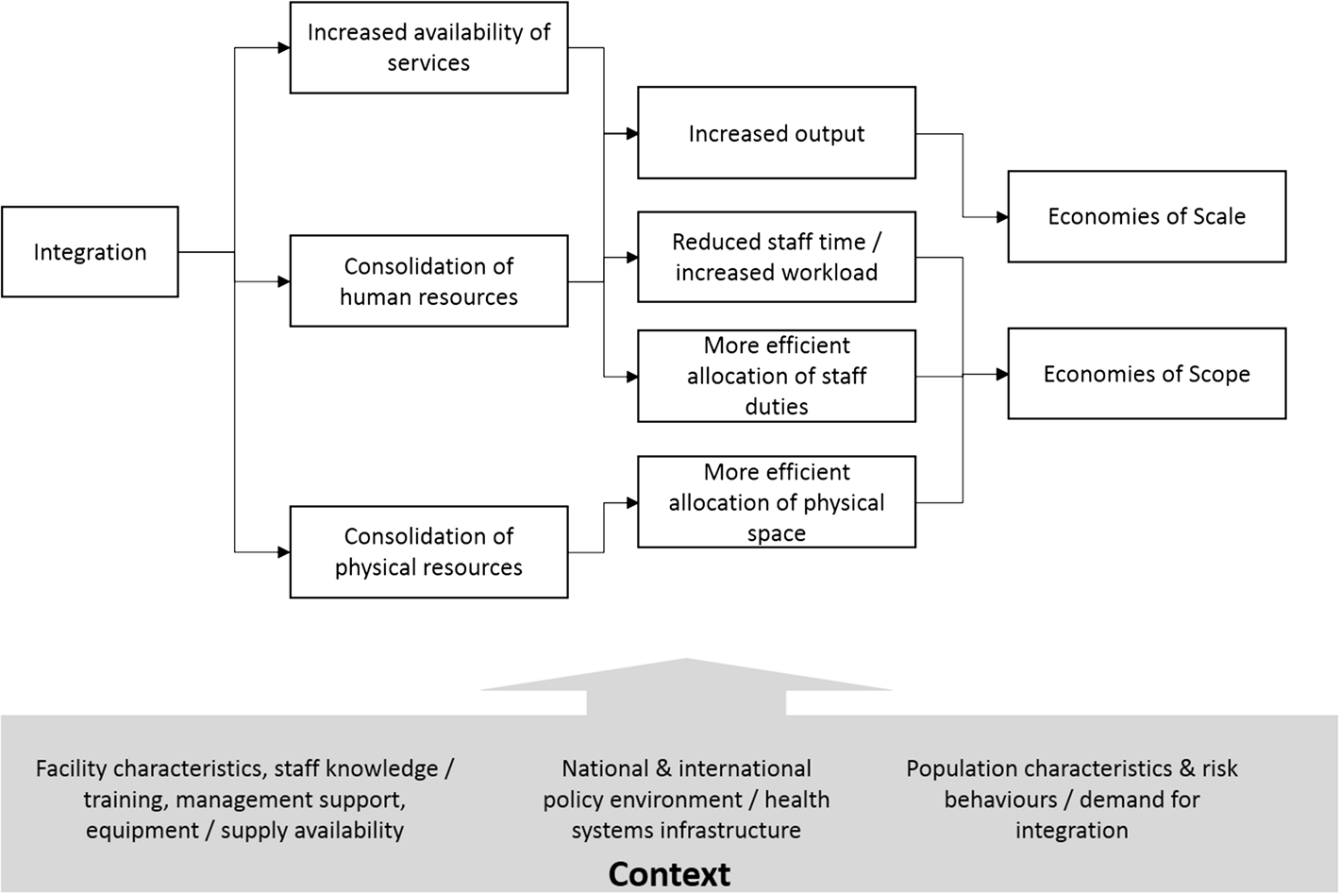
Economic impact of integration.

The theoretical economic considerations for HR integration are associated with two economic concepts surrounding efficiency in health service delivery (Figure 1). First, integration may result in ‘economies of scale’, or cost savings through an increase in the number of services delivered with the same level of staff. Integration can lead to increased scale as it can enable staff to offer additional services to clients (for example PITC). Integration can also help to make health service users more aware of what is offered or reduce other barriers to use - such as stigma - potentially leading to increased demand for services [23].

Second, it is hypothesized that integration can also result in ‘economies of scope’, or reductions of costs through joint production of goods/services. For example, an integrated service delivery model could reduce the number of times necessary to perform basic tasks such as height and weight measurement for a client receiving a number of different services, reducing the staff time required per patient. The effect of HR consolidation may be larger for certain combinations of services. For example, FP may be more naturally delivered together with PITC, as counselling for both are easily combined.

### Data collection and analysis

To explore whether these hypothesized benefits can be realized in practice, we conducted a descriptive analysis of HR integration, staff time utilization and workload in all 40 Integra project sites in Kenya and Swaziland, using a three stage approach. The first stage in our analysis was to examine the extent of HR integration, and to describe its place within the broader context of HIV/SRH integration (Figure 1). We focused on an indicator of HR integration measured as ‘the range of services provided per SRH/HIV staff member’, and calculated as the total number of non-core SRH/HIV services provided by staff working within the MCH unit (taking a value of one to five), divided by the total staff full time equivalency (FTE) allocated to these services at the facility level. The total number of staff FTE available in the unit during the baseline and endline time periods was sourced from facility records and observations, and confirmed through interviews.

In order to place HR integration within the wider context of organizational change, we also considered three further indicators of integration (Mayhew S, Ploubidis G, Church K, Obure CD, Zhou W, Sweeney S, Birdthistle I, DuPreez NF, Watts C, Warren CE, Vassall A, Integra Initiative: Innovation in measurement of service integration over time: the Integra Index of sexual and reproductive health integration, submitted). The first two of these measures describe a) the range of services provided at the facility level, and b) the range of services provided within the MCH/FP unit. A service was considered ‘provided’ if more than ten visits were recorded in a year. The other indicator represents integration of physical resources, measured as the ‘range of services provided per room’. Improvements in these other elements of integration were evaluated against improvements in HR integration in order to determine whether there was any interaction across the various aspects of integration.

Stage two in the analysis involved an adaptation of basic methods from the WHO’s Workload Indicators of Staffing Needs (WISN) to estimate a workload ratio similar to the WISN ratio - measured as the ratio of the actual staffing levels to the estimated staffing requirement for certain services [24,25]. We estimated the time required to deliver different services through a mixed methods approach to staff time observation; this is an improvement on the typical WISN methodology which uses expert opinion on timing of health services and may not reflect real practice [26]. We used these time observations combined with detailed service statistics to estimate the total staff FTE required to deliver services in each facility. Our estimates conservatively assume 220 working days per year accounting for national holidays and leave time, and assume 33% of this time to be taken by administrative duties, trainings and so on - leaving 70,752 annual minutes per clinical staff FTE for direct patient care. For some services, including HIV counselling and testing and HIV care and treatment, we also considered the time of technical staff such as lab technicians and lay counsellors. We divided actual staffing levels by the estimated staff FTE required to deliver services within each facility to obtain the workload ratio. A workload ratio greater than one indicates some down time for staff members. As the ratio reaches one, the estimated time taken to deliver outputs is equal to the staff time available for patient visits within a facility, while a ratio less than one would indicate that staff are likely overworked (that is the time required to attend patients is greater than the staff time available).

Finally, stage three of the analysis involved an examination of the relationship between HR integration and staff workload - both for individual services, and at the facility level. We conducted a bivariate categorical analysis of workload ratios in facilities with high- and low-integration. We first identified facilities with the top 20% of HR integration scores for their facility type (‘more integrated’), and compared these against the bottom 80% (‘less integrated’). As the extent of integration was different depending on facility type, the cut-off point between ‘more integrated’ and ‘less integrated’ facilities ranged from an HR integration score of 0.82 at Public Health Units, to 2 at Health Centres, and 3 at remaining facility types. We also identified the facilities with the top 20% in positive change in HR integration from baseline to endline for their facility type (‘most improved’), and compared these with the remaining facilities (‘least improved’). The cut-offs for most/least improved facilities were also stratified by facility type, with the greatest improvement seen at Health Centres and the lowest at District Hospitals and Sub-District Hospitals. We compared the workload ratio between these categories of HR integration to estimate any impact integration may have on staff workload.

### Data sources

Data on facility organization, staff time and workload was collected using two instruments: a semi-structured interview and records review tool, and a costing instrument. Both instruments were pretested in field sites and revised before implementation. Relevant data was collected at both baseline and endline.

In order to estimate the staff time required to deliver different services, we took a mixed methods approach in observation of staff time during endline data collection; this included key informant interviews with staff, followed by one week of direct observation by researchers to time consultations and concurrent time sheets completed by facility staff members. This was then followed up by a confirmatory interview with the staff member at the end of the observation period to discuss any discrepancies between data sources.

To estimate the number and range of services provided, we collected detailed output data on the total number of visits per year for different services from routine standardized monitoring registers kept within the facility.

All data collection was conducted by two researchers and quality controlled by a third researcher. Ethical approval for data collection was obtained from the London School of Hygiene & Tropical Medicine, Population Council Institutional Review Board, Kenya Medical Research Institute National Ethical Review Committee, and Swaziland Scientific Review Board. Written informed consent was obtained for all Integra study activities.

### Data analysis

Data was entered into standardized Excel worksheets, and analyzed using Excel (Microsoft Corp., Redmond, WA, USA) and Stata 13 [27]. For our stage one analysis, Fisher’s exact tests were used to examine the association between improvements in HR integration and other aspects of structural integration (including physical resource integration and range of services available). For stage two, we estimated the workload ratio according to service and facility type using the mean time observed per service across all facilities. We also estimated the impact of increasing the estimated staff time per service to the upper bound of the 95% confidence interval from our observations. Finally, we explored differences in workload estimates between HR integration category in stage three of our analysis, testing for significance at the *P* < 0.05 and *P* < 0.10 levels using Student’s *t*-tests, assuming unequal variance where applicable.

## Results

### Expansion of scope

The mean HR integration indicator, measuring the range of services per SRH/HIV staff member, was 1.75 services at baseline (median 2.0) and 1.69 services at endline (median 1.9). The only service consistently provided by SRH/HIV staff across all facilities was FP. CD4 count services were not delivered within the MCH unit at any facility. Twelve of the 40 facilities had an improvement in HR integration from baseline to endline. The HR integration indicator decreased from baseline to endline in 15 facilities, and 13 had no change (see Appendix 1).

**Figure 2:**
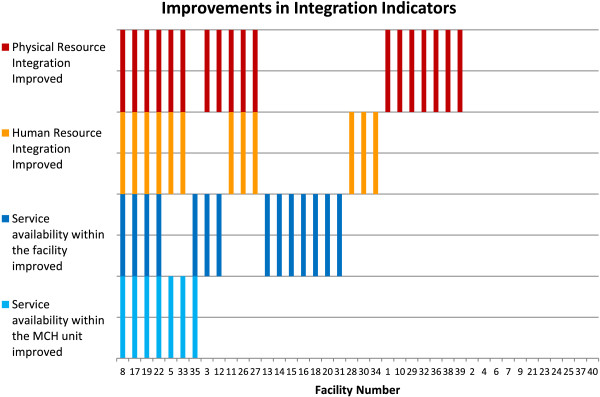
Improvements in human resource (HR) integration from baseline to endline.

Improvements in HR integration were significantly associated with some improvements in the wider context of integration (Figure 2). Facilities which improved HR integration also tended to improve physical resource (room) integration (*P* = 0.02) and availability of services within the MCH unit (*P* = 0.01). Improvements in HR integration were not associated with improved service availability at the facility level. We also found no trends in either levels or changes in integration that were consistent across facility type or ownership. Levels and changes in the four integration indicators are discussed more broadly in Appendix 1.

### Staff time and workload

The time taken to deliver an HIV/SRH outpatient visit during endline observations varied widely. On average, the service taking the longest time to deliver was PNC (mean 14.32 minutes), followed by STI (mean 11.44 minutes). The distributions of time taken for a number of services were heavily left-skewed, accounting for a minority of complicated cases with longer consultation times. The mean and median time observed, along with the upper-bound and lower-bound estimates, are presented by service type in Table 1.

**Table 1:** Staff time observed per consultation

	**Number of observations**	**Number of minutes per consultation**				
		**Mean**	**Median**	**95% CI**		
FP visit	280	8.26	6.00	7.48	to	9.04
PNC visit	98	14.43	15.00	12.78	to	16.09
Ca Cervix screening visit	20	7.40	6.50	5.33	to	9.47
STI visit	9	11.44	11.00	8.89	to	14.00
HIV counselling and testing visit	33	9.74	7.00	7.14	to	12.34
HIV care and treatment visit	62	10.15	5.00	7.48	to	12.81
Other MCH/OPD visit	172	8.13	4.50	6.78	to	9.48

Observations conducted during endline data collection.

FP, family planning; MCH/OPD, maternal and child health/outpatient department; PNC, postnatal care; STI, sexually transmitted illness.

Table 2 shows SRH/HIV service utilization and staff available at baseline and endline, by facility type. Service utilization increased on the whole from baseline to endline. The total number of SRH/HIV outpatient visits varied according to facility size, from 213 to 7,169 patient visits per year at baseline, and from 336 to 11,995 patient visits per year at endline. Facility staffing levels also increased in both countries from baseline to endline; with 32 facilities increasing the staff FTE available for SRH/HIV services. On average, the greatest increases in staff FTE available were observed for HIV care and treatment (from an average of 3.31 FTE at baseline to 5.99 FTE at endline), and VCT services (from 1.6 to 2.55 FTE). The mean number of outpatient visits per staff member per day was 17 at baseline and 15 at endline, however this ranged from 2 to 22 patients/FTE/day at baseline and from 2 to 37 patients/FTE/day at endline. Hospitals and public health units generally had greater staff FTE available than smaller rural facilities, however there was no observable trend for the number of patient visits per staff member per day by country or facility type.

**Table 2:** Workload indicators

		**Number of facilities**		**Mean annual patient visits**		**Mean staff FTE available**		**Mean workload ratio**	
		**Baseline**	**Endline**	**Baseline**	**Endline**	**Baseline**	**Endline**	**Baseline**	**Endline**
District Hospital (n = 5)	FP	5	5	6,350	5,438	1.41	1.41	1.69	1.92
	PNC	1	2	426	926	0.14	0.18	1.42	0.80
	Ca Cervix screening	3	5	230	265	0.05	0.07	1.62	2.34
	STI	3	2	206	561	0.06	0.10	2.04	1.14
	HIV care and treatment	2	2	3,912	10,161	6.45	11.06	12.81	5.81
	PITC	5	5	1,080	1,472	0.41	0.61	2.32	2.16
	VCT	5	5	2,432	6,006	2.26	4.27	6.49	8.95
Health Centre (n = 17)	FP	17	17	1,756	2,388	0.65	1.09	2.45	3.71
	PNC	11	12	378	498	0.36	0.27	2.80	1.83
	Ca Cervix screening	3	5	151	141	0.10	0.18	5.40	14.05
	STI	7	4	131	858	0.03	0.27	0.85	3.70
	HIV care and treatment	8	10	7,343	8,187	4.01	6.84	5.96	12.48
	PITC	14	15	485	537	0.19	0.32	2.75	3.70
	VCT	9	6	889	1,625	0.87	1.09	11.88	6.25
Hospital (n = 2)	FP	2	2	5,188	6,508	2.17	1.79	2.32	2.12
	PNC	1	1	1,985	2,319	1.43	2.45	3.53	4.90
	Ca Cervix screening	2	2	264	781	0.06	0.67	1.91	8.17
	STI	2	2	31	54	0.01	0.03	1.89	2.85
	HIV care and treatment	1	1	38,772	70,605	16.23	28.80	2.37	2.64
	PITC	2	2	2,376	1,078	1.29	1.82	4.16	8.40
	VCT	2	2	2,930	2,290	4.38	2.57	6.60	8.16
Public Health Unit (n = 2)	FP	2	2	12,634	16,314	2.63	2.90	1.47	1.29
	PNC	2	2	3,193	2,524	0.74	2.70	1.08	5.04
	Ca Cervix screening	1	1	110	19	0.02	0.00	1.46	1.81
	STI	0	2	-	214	-	0.04		2.46
	HIV care and treatment	1	2	4,878	457	3.46	3.65	4.58	49.75
	PITC	2	1	1,278	2,681	0.66	0.19	3.31	0.51
	VCT	1	1	1,357	1,036	0.42	2.52	2.10	16.89
SRH Clinic (n = 8)	FP	8	8	3,889	5,386	0.91	2.12	1.88	2.19
	PNC	7	7	160	187	0.14	0.08	4.41	0.80
	Ca Cervix screening	8	8	588	714	0.19	0.31	4.03	29.34
	STI	8	7	454	1,051	0.25	0.21	3.74	1.84
	HIV care and treatment	7	7	468	809	0.82	1.70	13.58	10.44
	PITC	7	7	802	386	0.20	0.22	2.38	2.53
	VCT	8	7	1,936	5,308	1.59	3.19	4.92	5.00
Sub-District Hospital (n = 6)	FP	6	6	1,974	2,386	0.63	0.62	2.83	2.41
	PNC	3	3	229	1,162	0.12	0.49	1.77	2.69
	Ca Cervix screening	2	5	26	85	0.01	0.02	3.48	4.51
	STI	4	1	46	24	0.02	0.00	1.89	0.70
	HIV care and treatment	3	3	341	1,636	0.82	3.71	15.51	17.82
	PITC	5	5	487	2,018	0.16	0.46	13.94	3.66
	VCT	6	3	590	1,717	1.44	1.11	25.63	8.78

FP, family planning; FTE, full time equivalency; PITC, provider-initiated HIV testing and counselling; PNC, postnatal care; STI, sexually transmitted illness; VCT, voluntary HIV counselling and testing.

**Figure 3:**
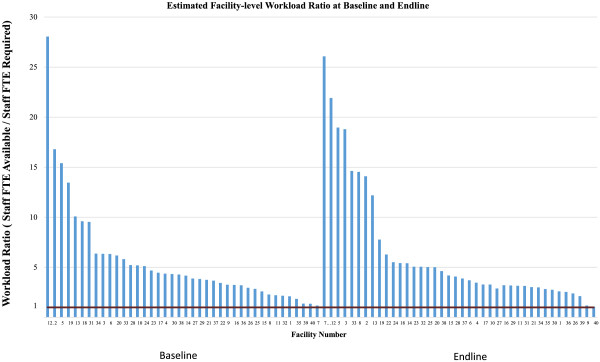
Estimated facility-level workload ratio at baseline and endline.

The estimated number of staff required to deliver services also varied widely, both by service and facility type (see Tables 3 and 4). When evaluating workload by service type, the highest average workload ratio was for VCT (11.22) at baseline and for HIV Care (14.60) at endline - indicating high excess capacity for these services across all facility types. STI on average had the lowest workload ratio at baseline (2.22) and PNC had the lowest ratio at endline (1.93). Several individual services had workload ratios less than one indicating insufficient capacity; this was most common for PNC (in ten facilities at baseline, and twelve facilities at endline), followed by PITC (in eight facilities at baseline and three at endline) and STI (in four facilities at baseline and six at endline).When we examined overall workload at the facility level, including other outpatient and MCH services, the workload ratio was high, but again with a substantial variation by facility (Figure 3). The mean facility-level ratio was 5.67 at baseline (SE 0.81), and 6.53 at endline (SE 0.97). No facilities had a ratio less than one at baseline or at endline when estimated using the mean staff time observed. Using the upper bound observations for all services reduced the mean facility-level workload ratio slightly to 4.72 at baseline and 5.40 at endline, with two facilities indicating a ratio less than one at endline (0.91 and 0.92).

**Table 3:** Workload and staffing, by human resource (HR) integration category ‘more/less integrated’

	**‘Less integrated’ facilities (n = 58)**			**‘More integrated’ facilities (n = 22)**		
	**Mean**	**Mean**	**Mean workload ratio (low/high estimates)**	**Mean**	**Mean**	**Mean workload ratio (low/high estimates)**
	**Staff FTE available**	**Staff FTE required**		**Staff FTE available**	**Staff FTE required**	
Ca Cervix screening	0.06	0.02	10.98 (8.58 to 15.25)	0.10	0.03	6.79 (5.30 to 9.42)
FP	0.91	0.42	2.66 (2.43 to 2.93)	0.99	0.54	2.15 (1.96 to 2.37)
HIV care and treatment	3.21	0.87	12.09 (9.58 to 16.40)	0.45	0.03	15.86 (12.57 to 21.52)
PITC	0.33	0.09	4.65 (3.67 to 6.34)	0.22	0.14	2.19^a^ (1.73 to 2.98)
PNC	0.37	0.13	2.48 (2.22 to 2.80)	0.17	0.13	2.03 (1.82 to 2.29)
STI	0.05	0.03	2.68 (2.19 to 3.45)	0.09	0.06	1.63^b^ (1.33 to 2.09)
VCT	1.10	0.20	10.08 (7.96 to 13.76)	1.66	0.31	8.32 (6.57 to 11.35)
Other MCH/OPD service	5.44	1.09	8.39 (7.19 to 10.06)	6.19	1.70	14.69 (12.59 to 17.61)
Total facility	**10.94**	**2.62**	**5.61 (4.62 to 7.15)**	**9.34**	**2.87**	**6.72 (5.63 to 8.35)**

^a^difference from ‘less integrated’ group significant at the *P* < 0.10 level (*t* = 1.79, *P* = 0.078).

^b^difference from ‘less integrated group significant at the *P* < 0.05 level (*t* = 2.05, *P* = 0.047).

FP, family planning; FTE, full time equivalency; MCH/OPD, maternal and child health/outpatient department; PITC, provider-initiated HIV testing and counselling; PNC, postnatal care; STI, sexually transmitted illness; VCT, voluntary HIV counselling and testing.

**Table 4:** Workload and staffing, by human resource (HR) integration category ‘least/most improved’

	**Mean staff FTE available**		**Mean staff FTE required**		**Mean workload ratio (low/high estimates)**	
	**Baseline**	**Endline**	**Baseline**	**Endline**	**Baseline**	**Endline**
‘Least improved’ facilities						
(n = 32)						
Ca Cervix screening	0.05	0.09	0.02	0.03	3.08 (2.41 to 4.28)	9.72 (7.60 to 13.50)
FP	0.82	0.99	0.43	0.53	2.06 (1.88 to 2.27)	2.16 (1.97 to 2.38)
HIV care and treatment	2.07	3.38	0.57	1.01	12.38 (9.81 to 16.80)	13.15 (10.42 to 17.84)
PITC	0.25	0.38	0.10	0.12	4.56 (3.60 to 6.22)	3.43 (2.71 to 4.68)
PNC	0.24	0.50	0.12	0.15	2.56 (2.29 to 2.88)	2.11 (1.89 to 2.38)
STI	0.06	0.06	0.02	0.06	1.96 (1.60 to 2.53)	2.37 (1.93 to 3.05)
VCT	1.11	1.35	0.16	0.31	9.60 (7.57 to 13.09)	7.33 (5.79 to 10.00)
Other MCH/OPD service	4.85	6.05	1.15	1.41	10.64 (9.13 to 12.76)	9.78 (8.38 to 11.72)
Total facility	**8.80**	**11.71**	**2.35**	**3.14**	**5.50 (4.58 to 6.89)**	**5.93 (4.91 to 7.50)**
‘Most improved’ facilities						
(n = 8)						
Ca Cervix screening	0.05	0.10	0.01	0.01	4.82 (3.76 to 6.69)	24.66 (19.27 to 34.24)
FP	0.72	1.40	0.33	0.36	2.99 (2.73 to 3.30)	5.31 (4.85 to 5.87)
HIV care and treatment	2.12	4.89	0.66	0.63	2.27 (1.80 to 3.08)	20.42 (16.18 to 27.71)
PITC	0.22	0.25	0.06	0.09	3.20 (2.53 to 4.37)	3.37 (2.66 to 4.60)
PNC	0.28	0.13	0.06	0.11	5.11 (4.58 to 5.77)	1.17^a^ (1.05 to 1.32)
STI	0.07	0.04	0.01	0.03	5.08 (4.15 to 6.53)	1.93 (1.57 to 2.48)
VCT	1.20	1.50	0.15	0.25	17.99 (14.20 to 24.54)	7.54 (5.95 to 10.29)
Other MCH/OPD service	5.88	6.93	1.07	1.28	9.72 (8.33 to 11.65)	9.80 (8.40 to 11.75)
Total facility	**9.64**	**13.36**	**2.09**	**2.40**	**6.37 (5.26 to 8.08)**	**8.94 (7.34 to 11.43)**

^a^difference from baseline significant at the *P* < 0.05 value (*t* = 2.45, *P* = 0.044).

FP, family planning; FTE, full time equivalency; MCH/OPD, maternal and child health; PITC, provider-initiated HIV testing and counselling; PNC, postnatal care; STI, sexually transmitted illness; VCT, voluntary HIV Counselling and testing.

### HR integration and workload

Tables 3 and 4 present service and facility workload ratios according to HR integration category from stage three of our analysis. Thirteen facilities at baseline and nine at endline were classified into the ‘more integrated’ group (top 20%). The ‘more integrated’ facilities delivered an average of 2.7 services per staff member, while ‘less integrated’ facilities delivered an average of 1.3 services per staff member. Thirty-two facilities were classified into the ‘least improved’ group, with an average decline in HR integration from baseline to endline of -0.29 services per staff member (from 1.85 to 1.56). In contrast, the eight ‘most improved’ facilities had an average improvement in HR integration of 0.84 services per staff member (from 1.13 to 1.96) (see Appendix 1). There was no significant difference between the ‘most improved’ and ‘least improved’ facilities in changes in staff numbers between baseline and endline. Similarly, there was no significant difference in overall staff numbers between ‘more integrated’ and ‘less integrated’ facilities.

Comparing ‘more integrated’ against ‘less integrated’ facilities (Table 3) reveals that the workload ratio for several individual services appear lower in the ‘more integrated’ group; this was only significant for PITC (*t* = 1.79, *P* = 0.078) and STI (*t* = 2.05, *P* = 0.047). The overall facility-level workload ratio was not significantly different between the two groups.

Comparing changes in integration and workload over time shows a different picture (Table 4). First, it is important to note that on average, ‘most improved’ facilities had relatively lower workload ratios at baseline than ‘least improved’ facilities; this was true for all services except HIV care and treatment. Thereafter, the increase in staff FTE available for HIV Care was greatest for the ‘most improved’ group. On average ‘most improved’ facilities increased staff available for HIV care by 5.07 FTE while ‘least improved’ facilities increased by only 2.23 FTE. In contrast, ‘most improved’ facilities on average had a decrease in total staff time available for other services such as PITC, PNC, STI, and VCT (see Table 4). In this context, when we compared changes in integration and workload over time, some individual services had significantly greater decrease in the workload ratio in the ‘most improved’ group than observed in the ‘least improved’ group. For example, we found a significant decrease in the average workload for PNC in ‘most improved’ facilities (*t* = 2.45, *P* = 0.044), but we found no significant change in ‘least improved’ facilities.

More broadly however, improvements in HR integration were not significantly associated with overall increases in facility-level workload. Facilities in both groups had an average facility-level increase in the workload ratio from baseline to endline.

## Discussion

This paper has provided a descriptive analysis of HR integration and staff workload across 40 facilities in Kenya and Swaziland. Our results indicate that HR integration was more likely to be improved in facilities which also improved other elements of integration, such as availability of services within the MCH unit and integrated use of physical space. Furthermore, while we there was no overall relationship between integration and workload at the facility level, certain services had a significantly lower workload ratio in more integrated facilities. These results suggest that HIV/SRH integration may be most influential on staff workload for PITC, PNC and STI services - particularly where HIV care and treatment services are being supported with extra staffing. Our findings therefore suggest that there may be potential for improving use of excess capacity through integration, but that overall the pace of increase in productivity is slow.

Our findings should be viewed in the context of a number of studies that have highlighted the urgent need for improving staffing and skill mix, especially in light of the demands on health services in the context of the HIV epidemic [14,20,28]. However, our findings are in line with excess capacity found in a number of settings [29-32]. The differences we found in workload between different services within facilities imply that this under-utilization of staff is not solely a site location-related issue, and can be improved through re-allocation of staff duties across services within sites. Similar imbalances in allocation of staff duties within facilities have been found in Uganda [33] and South Africa [29].

However facility-level workload ratios also varied substantially between facilities, from 1.18 to 28.05 at baseline and from 1.07 to 26.08 at endline. No facilities were found to be ‘overworked’ at baseline or endline given our assumptions and calculations. This was observed despite an overall increase in the number of HIV/SRH outpatient visits for most services over the study period, as in both countries there was a corresponding increase in staff time available. Some of these increases, in particular increased staffing of HIV-related services, may have come at the cost of reductions of staff available for other services such as PNC, and lead to greater imbalances in staff workload within a facility.

Although integration may be a route to streamlining HR use on the whole, integration was not achieved uniformly across facilities in either country, suggesting implementation challenges. HR integration was positively associated with other aspects of integration, including service availability and physical integration of services, suggesting that various aspects of integration go hand-in-hand. For example, investments in physical infrastructure and drug/supply availability are perhaps required before proceeding with HR integration. We therefore recommend that efforts to integrate should be preceded by some investigation at the facility level regarding capacity to integrate services. Moreover, planners should be flexible in design, as there is no ‘one-size fits all’ solution to integrating.

There are some indications from qualitative work that integration can lead to an improvement in quality of care, as providers may be able to deliver a more well-rounded service to patients [11]. However, in other cases, high workloads can negatively impact service quality [34]. Although our workload estimates indicate excess capacity this does not mean that staff are not under stress at all periods in the day. This is especially true in settings where client load is higher in the mornings, with demand falling in the afternoon. In this context, we therefore recommend careful monitoring and evaluation of workloads as integration progresses on a site-by-site basis.

It also should be noted that integration of HIV services into primary health care or other services typically delivered by lower-paid cadres (for example, nurses) has been proposed by some as a vehicle to reduce the reliance on higher-paid members of staff (such as clinical officers or doctors), enabling them to deal with more complicated cases [35-37]. However, care has to be taken, as at some lower-level health facilities there may not be sufficient demand for these more complex services. For example, in many cases, we observed the workload for SRH services to be much higher than that of HIV care and treatment, or other outpatient services typically delivered by a clinical officer or other higher-paid staff members. There is a risk that integration could lead to further overworking those lower-paid staff members, suggesting that integration efforts should be placed within the broader picture of HR planning and management. This may mean that additional staff training is required at the facility level in order to ensure that integration through task shifting does not result in a reduction of service quality.

Finally, the above findings and interpretation need to be seen in the context of a number of limitations. Although this is the largest study to date evaluating integration, we were limited in any multivariate analysis by the relatively small sample size. Furthermore, the nature of time use data is such that there is a risk of bias in all staff time observations [38]. Workload ratio estimates are relatively sensitive to the staff time observed per service. We tried to minimize bias through triangulation of several data collection approaches; however, it is possible that we have under- or over-estimated the time taken to deliver services. Similarly, as our utilization data was collected retrospectively from routine data registers, it is possible that services were delivered but not recorded in some facilities; this would under-estimate utilization, and thus workload. This under-reporting may not be consistent across service types. Of particular concern is STI utilization data, which was not consistently recorded across facilities. In addition, all staff time observations used in our calculations were recorded at the endline of the Integra study. Although staff were observed at baseline, observations were not systematically recorded at each of the study facilities, largely because few facilities consistently provided integrated services at baseline. We also did not account for the time differences between first visits and revisits for services such as FP or PNC, where a first visit can take much more time than a follow-up visit. For these particular services, we observed a large number of consultations and, therefore, capture some of this timing variability within our calculations. Finally, our recommendations are made from the provider perspective; and we take little account of how patients may value different visit times and experience facilities with relatively high workloads [39].

## Conclusion

In the context of the growing HIV/AIDS epidemic and the current economic climate, countries are increasingly interested in rationalizing the use of human resources for health care, with SRH/HIV integration seen as a key policy vehicle for achieving this. The need to ensure that scarce human resources are used efficiently must also be weighed against the risk of burnout for health workers who may feel overworked. This descriptive analysis explores the effect of HIV/SRH integration on staff workload through economies of scale and scope in high- and medium-HIV prevalence settings. We find some evidence to suggest that there is potential to improve productivity through integration, however with some significant challenges, and the pace of productivity gain slow. We recommend that any efforts to implement integration are fully assessed in the broader context of HR planning both within and between facilities to understand the impact on different cadres and minimize displacement effects in order to ensure that neither staff nor patients are negatively impacted by integration policy.

## Appendix 1

### Structural integration

At the facility level, there were considerable changes in all four indicators of structural integration in both directions. Twenty-nine facilities saw an improvement in at least one of the four measures of resource integration from baseline to endline, while twenty-two saw a decline in at least one of the four measures. We found no trends in either levels or changes in integration that were consistent across facility type or ownership (Table 5).The number of HIV and other non-core services available within the MCH/FP unit (taking a possible value one to five) was the least common improvement - taking place in only seven facilities. This improvement did not take place without other indicators of integration in place. Availability of total core and non-core services available in the facility (taking a possible value one to eight) improved in fifteen facilities (Figure 2). The most common improvement was in the number of non-core RH/HIV services provided in each consultation room in the MCH/FP unit (possible value one to five), with eighteen facilities (eleven urban and seven rural facilities) improving their physical resource integration from baseline to endline.

**Table 5:** Integration indicators by health facility characteristics

	**Service Availability in MCH/FP Unit (out of 5)**		**Service Availability in Facility (out of 8)**		**Human Resources Integration (out of 5)**		**Physical Resources Integration (out of 5)**	
	**Baseline**	**Endline**	**Baseline**	**Endline**	**Baseline**	**Endline**	**Baseline**	**Endline**
Country								
Kenya (n = 30)	2.23	2.3	6.1	6.56	1.88	1.93	1.28	1.29
Swaziland (n = 10)	2.2	2.3	6.7	7	1.36	1.01	1.15	1.18
HR integration								
More integrated (top 20%)	2.69^b^	3.11^c^	6.38	7.11^b^	2.62^d^	2.88^d^	1.58	1.09^b^
Less integrated (bottom 80%)	2	2.06	6.19	6.52	1.33	1.34	1.10	1.86
Change in HR integration								
Most changed (top 20%)	1.75	2.50	6.00	7.00	1.17	2.01^a^	0.67	1.36^a^
Least changed (bottom 80%)	2.34	2.25	6.31	6.56	1.90	1.61	1.40	1.24
Facility type								
Hospital (n = 2)	3	3	8	8	2.77	1.79	0.98	0.59
District Hospital (n = 5)	2.2	2.4	7.8	7.82	1.94	2.34	1.37	0.9
Sub-District Hospital (n = 6)	2	1.84	6.33	6.36	2	1.75	1.16	1.03
Health Centre (n = 17)	1.41	1.52	5.35	6.18^a^	1.15	1.21	0.71	0.95^a^
Public Health Unit (n = 2)	2.5	3	5.5	6.5	0.77	0.35^a^	0.88	0.8
SRH Clinic (n = 8)	3.87	3.87	6.87	6.87	2.72	2.54	2.58	2.6
Location								
Rural (n = 23)	1.57	1.61	5.61	6.24^a^	1.37	1.35	0.83	0.97
Urban (n = 17)	3.11	3.23	7.12	7.24	2.26	2.13	1.83	1.64
Ownership type								
Private (n = 8)	3.87	3.87	6.87	6.87	2.72	2.54	2.58	2.6
Public (n = 32)	1.81	1.91	6.09	6.63	1.51	1.47	0.92	0.92

^a^difference from baseline significant at the *P* < 0.10 level.

^b^difference from 'less integrated' group significant at the *P* < 0.10 level.

^c^difference from 'less integrated' group significant at the *P* < 0.05 level.

^d^difference from 'less integrated' group significant at the *P* < 0.00 level.

CaCx screening was the service most commonly introduced to the MCH unit, with seven facilities adding screening services from baseline to endline. Four facilities also introduced PITC and four facilities introduced STI services within the MCH unit. One additional facility added PITC as a stand-alone service, and three facilities began providing HIV Care and treatment outside the MCH unit. Changes in service availability outside the MCH unit were not significantly associated with HR integration. Several of the facilities which added CaCx, HIV care, or PITC as described above also dropped a different service. The service most commonly dropped was STI (twelve facilities), followed by VCT (six facilities) and PITC (four facilities). No facilities provided CD4 count testing within the MCH unit, either at baseline or at endline.

## Abbreviations

SRH: sexual and reproductive health; HR: human resources; PITC: provider-initiated HIV testing and counselling; PNC: postnatal care; MCH: maternal and child health; FP: family planning; ANC: antenatal care; STIv: sexually transmitted infections; VCT: voluntary HIV counselling and testing; CaCx: cervical cancer screening; ART: antiretroviral therapy; FTE: full time equivalency; WISN: Workload Indicators of Staffing Needs; OPD: outpatient department.

## Competing interests

The authors declare that they have no competing interests.

## Authors’ contributions

All authors provided editorial input, contributed to subsequent drafts of the manuscript and reviewed the final version prior to submission. SS was involved in data collection, analysis, and interpretation of data and drafted the paper. CDO was involved with the study design, data collection, and assisted with the analysis. FTP contributed to the study design. VD was involved in data collection. CMI contributed to the study design, and was also involved in the data collection. EM and ZN contributed to the implementation of the study. CEW, SM and CW were involved in the overall conceptual design and implementation of the project and contributed to the overall revision of this manuscript. AV contributed to the study design and supervised data collection and analysis, assisted in interpretation of the study results and contributed to the drafting of the manuscript. All authors read and approved the final manuscript.
